# Impact of Diabetes and Smoking on Mortality in Tuberculosis

**DOI:** 10.1371/journal.pone.0058044

**Published:** 2013-02-28

**Authors:** George W. Reed, Hongjo Choi, So Young Lee, Myungsun Lee, Youngran Kim, Hyemi Park, Jongseok Lee, Xin Zhan, Hyeungseok Kang, SooHee Hwang, Matthew Carroll, Ying Cai, Sang-Nae Cho, Clifton E. Barry, Laura E. Via, Hardy Kornfeld

**Affiliations:** 1 Department of Preventive and Behavioral Medicine, University of Massachusetts Medical School, Worcester, Massachusetts, United States of America; 2 International Tuberculosis Research Center, Changwon, Republic of Korea; 3 Department of Microbiology, Yonsei University College of Medicine, Seoul, Republic of Korea; 4 Information Services Department, University of Massachusetts Medical School, Worcester, Massachusetts, United States of America; 5 National Masan Tuberculosis Hospital, Changwon, Republic of Korea; 6 Tuberculosis Research Section, Laboratory of Clinical Infectious Disease, National Institute of Allergy and Infectious Diseases, Bethesda, Maryland, United States of America; 7 Department of Medicine, University of Massachusetts Medical School, Worcester, Massachusetts, United States of America; Fundació Institut d’Investigació en Ciències de la Salut Germans Trias i Pujol. Universitat Autònoma de Barcelona. CIBERES, Spain

## Abstract

**Background:**

Diabetes mellitus is a risk factor for tuberculosis (TB) disease. There is evidence that diabetes also influences TB severity and treatment outcomes but information is incomplete and some published results have been inconsistent.

**Methods:**

A longitudinal cohort study was conducted at the National Masan Tuberculosis Hospital in the Republic of Korea. Subjects presenting with a first episode of TB or for retreatment of TB were followed from enrollment through completion of treatment. Demographic, clinical, and microbiological variables were recorded, along with assessment of outcomes. Results were compared in TB patients with and without diabetes or smoking history. Data were adjusted for gender, age, cohort, educational level and alcohol consumption.

**Results:**

The combined cohorts comprised 657 subjects. Diabetes was present in 25% and was associated with greater radiographic severity and with recurrent or relapsed TB. Diabetes and cigarette smoking independently increased the risk of death in the first 12 months after enrollment. Estimating the combined impact of diabetes and smoking yielded a hazard ratio of 5.78. Only 20% of diabetic subjects were non-smokers; 54% smoked ≥1 pack daily. In this cohort, the impact of diabetes on mortality was greater in patients younger than 50 years, compared to older patients.

**Conclusions:**

In this cohort of Korean patients, diabetes exacerbated the severity of TB disease. Diabetic subjects who smoked ≥1 pack of cigarettes daily were at particularly high risk of death from TB. Strategies to improve TB outcomes could productively focus resources for patient education and TB prevention on the vulnerable population of younger diabetics, particularly those who also smoke.

## Introduction

Tuberculosis (TB) remains a major cause of morbidity and mortality from infectious disease worldwide [1]. It is estimated that two billion people are presently infected with causal agent, *Mycobacterium tuberculosis*. Within the large pool of persons with latent TB infection (LTBI) there is a ∼5% lifetime risk of developing active TB disease. TB disease risk is influenced by inherited and acquired susceptibility factors. HIV/AIDS has long been appreciated as a major acquired risk factor, with relative risk estimated between 20 and 37 depending on the local state of the HIV epidemic [2]. Diabetes and exposure to cigarette smoke have more recently been identified as acquired risk factors for TB [3], [4]. While the TB risk conferred by diabetes is lower than HIV/AIDS, its high prevalence makes it comparable to HIV/AIDS in terms of global population-attributable TB risk [5].

In addition to increasing the risk for developing active TB there is evidence that diabetes is also associated with greater severity of TB disease. It has been linked to higher mortality, delayed sputum conversion, increased radiographic extent of disease and greater risk of recurrent TB after treatment [6]. While published evidence has generally supported the hypothesis that diabetes increases TB severity there have been inconsistencies in identifying certain associations, including delayed sputum conversion and increased risk for multi-drug resistant (MDR) TB, and differences in the estimated impact of diabetes on TB mortality. In many studies of TB and diabetes, data were not adjusted for potentially confounding variables and study populations tended to be small. To expand the base of knowledge on this topic we made use of data collected prospectively in a Natural History Study ongoing at the International TB Research Center and the National Masan Tuberculosis Hospital in the Republic of Korea. We aimed to evaluate the impact of diabetes on the clinical presentation, treatment response and outcomes of pulmonary TB. Our primary goal was to test the hypothesis that co-morbid diabetes increases the risk for adverse TB outcomes. Secondary goals included analyzing the combined effects of diabetes and smoking, and testing the impact of diabetes on TB outcomes specifically in the Korean population. There is potential for geographic and ethnic differences in the effects of diabetes on TB disease manifestations and outcomes that have not been adequately investigated. Such differences could arise due the relative virulence of locally prevalent *M. tuberculosis* clades, along with potential differences in host genetic susceptibility to TB and/or differences in the mechanisms and manifestations of type 2 diabetes in distinct populations [7], [8]. The Korean population is ethnically and genetically homogeneous, and nearly all *M. tuberculosis* isolates in Korea belong to the Beijing clade [9].

## Materials and Methods

### Study Design and Population

Participants were recruited from patients presenting to the National Masan Tuberculosis Hospital (NMTH) for the management of active TB disease. Subjects with a first-ever diagnosis of TB were assigned to cohort A while those previously treated for TB were assigned to cohort B. Inclusion criteria were males and females ≥20 years of age with symptoms compatible with active TB and a positive sputum smear for acid-fast bacilli (AFB). An additional inclusion criterion for cohort A was <30 days of antibiotic treatment for TB prior to enrollment. Pregnancy and HIV were exclusion criteria. Subjects with positive sputum smears at the time of enrollment but whose cultures subsequently grew only non-tuberculous mycobacteria (2/659) where withdrawn from the study. Subjects remained as inpatients at NMTH for an average of 165 days, according to usual clinical practice at that institution. Subjects discharged from the inpatient service at NMTH were subsequently followed in the NMTH outpatient clinic. Whenever possible, information on the outcomes for those no longer seen at NMTH was obtained from the subjects or their families as permitted by human studies protocols.

### Procedures and Investigations

Demographic data and clinical history were recorded by study nurses and routine laboratory data were collected from the NMTH medical record. The Natural History Study was not originally designed to focus on the influence of diabetes on TB outcomes but diabetic patients within cohorts could be reliably identified, allowing comparison to those without diabetes. Subjects were scored as diabetic if they described a history of this condition at the time of enrollment, if they were taking anti-diabetic medications prior to hospital admission, or if they had random blood glucose ≥200 mg/dL on two separate days in hospital. Chest X-rays were read by hospital radiologists and scored in blinded fashion according to a predetermined protocol used by the Korean Centers for Disease Control [10]. Radiographs were scored as showing minimal, moderately advanced, or far advanced TB, or having no TB disease evident. The presence or absence of cavitation and nodules was recorded, as were all lung lobes involved with disease.

Sputum for mycobacterial smear and culture was collected at months 0 1, 2, 4, and 6 of treatment according to usual clinical practice at NMTH and shared with the study staff. These tests were performed by standard methods in the laboratory of the International Tuberculosis Research Center, located adjacent to NMTH. Sputum was cultured on Ogawa solid media and in an MB/BacT liquid growth system. Drug sensitivity testing was performed on all positive cultures. Positive Ziehl-Neelsen stained sputum smears and solid media cultures were scored on a scale ranging from scanty to 4+. All subjects were inpatients at NMTH for at least the first 4 months of treatment and received directly observed therapy. Subjects with drug resistant TB remained in hospital for up to 7 months and, following discharge, were readmitted to the inpatient service if follow-up sputum smears were positive. Subjects having positive smear or culture at 6 months prompted a continuation of monthly sputum testing out to 12 months. Subjects positive beyond 12 months were monitored with cultures done at the discretion of the attending physicians. Treatment regimens in cohort B were individually tailored based on individual subject TB history and any available previous antimicrobial sensitivity testing data.

### Statistical Methods

Two-group comparisons of demographics at enrollment (cohort A vs. B) were made using chi-square tests for categorical measures and Student’s t-tests for comparison of means. Unadjusted rates of diabetes were compared among cohorts using Fisher’s exact tests. Odds Ratios (OR) for risk of diabetes were estimated and tested using logistic regression. Patients in cohorts A and B were matched using propensity score matching. The propensity (of being in cohort B) was estimated using age, gender, levels of drinking and smoking, education and occupation. Matching was carried out for all patients and separately with in age groups (age <50 and age ≥50). Estimates and tests of OR for the matched populations were generated using Generalized Estimating Equation logistic regression clustering on matched patients. Rank sum tests compared smear and culture scores; adjusted comparisons were made using linear regression. Time to clearance of sputum smear and culture was limited to cohort A. Unadjusted rates of time to clearance were estimated using Kaplan-Meier curves and adjusted time to clearance was modeled and tested using Cox regression. Rates of MDR-TB were compared using logistic regression models. Unadjusted mortality was estimated using Kaplan-Meier estimation. Cox regression models estimated unadjusted and adjusted hazard ratios. The association of diabetes and age was examined using lowess curves [11]. All analyses were carried out using Stata 12 (StataCorp, College Station, TX).

### Ethics

Individual participants in this study gave written informed consent under protocols approved by the Institutional Review Boards of the National Masan Tuberculosis Hospital (Republic of Korea) and the National Institute of Allergy and Infectious Diseases, National Institutes of Health (United States). Investigators from the University of Massachusetts Medical School were subsequently added to the National Masan Tuberculosis Hospital and National Institute of Allergy and Infectious Diseases protocols, but accessed only de-identified patient data.

## Results

### Cohort Description

Subjects were offered enrollment in the Natural History Study on presentation to NMTH for management of TB and stratified into those with first-ever TB (cohort A) or retreatment of TB (cohort B). Bacterial genotyping data on isolates obtained prior to enrollment were not available to determine whether individual subjects in cohort B represented treatment failure with persistently positive cultures, relapse of prior infection with the same strain after culture conversion or exogenous reinfection with a different *M. tuberculosis* strain. The rate of exogenous reinfection in the Korean population has not been reported. Using an algorithm based on the incidence of TB in a general population proposed and tested by Wang et al. [12], reinfection in Korea can be estimated in the range of 20–30%. Thus, most subjects in cohort B represented failure to clear initial infection or relapse with the same organism after clinical cure. Within the 657 total active TB cases, subjects in cohort B were younger, smoked less and had higher education than those in cohort A (Table 1).

**Table 1:** Demographic comparison of cohorts.*

	Cohort A	Cohort B	A vs. B
	n (%)**	n (%)**	p-value
**All subjects**	220	437	
**Female**	36 (16.4)	70 (16.0)	0.91
**Age**	46.6±15.0	43.5±13.2	0.005
**Smoking status**	213	413	<0.001
▪ Non-smoker	47 (22.1)	98 (23.7)	
▪ <1 pack per week	30 (14.1)	122 (29.5)	
▪ 1 pack per day	76 (35.7)	120 (29.1)	
▪ >1 pack per day	60 (30.8)	73 (17.7)	
**Drinking**	220	437	0.139
▪ None	80 (33.4)	192 (43.9)	
▪ ≤4 per week	57 (25.9)	115 (26.3)	
▪ 1 per day	15 (6.8)	29 (6.6)	
▪ >1 per day	68 (30.9)	101 (23.1)	
**Education**	206	421	0.007
▪ Elem. School	47 (22.8)	61 (14.5)	
▪ Middle School	40 (19.4)	115 (27.3)	
▪ High School	97 (47.1)	179 (52.5)	
▪ Univ/Prof	22 (10.7)	66 (15.7)	
**Occupation**	219	435	0.024
▪ Prof/office/health	16 (7.3)	54 (12.4)	
▪ Service sector	45 (20.6)	120 (27.6)	
▪ Laborer	81 (37.0)	120 (27.6)	
▪ Other	37 (16.9)	62 (14.3)	
▪ None	40 (18.3)	79 (18.2)	
**MDR-TB**	16 (8.3)	213 (40.6)	<0.01

* Cohort A, first ever TB diagnosis; cohort B, retreatment for TB.

** Includes only subjects where data on smoking, drinking, education and occupation were available.

**Figure 1:**
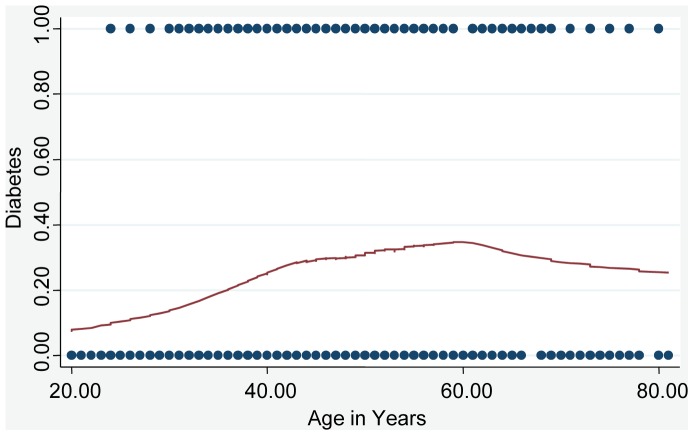
Non-linear association of diabetes and age. Lowess smoothing curve [10] fit to diabetes prevalence vs. age. A spline fit model (knot at age = 50) is significantly different from a linear fit (p<0.001).

Rates of diabetes were compared between cohort A (23.3%) and cohort B (25.2%), overall and within age groups <50 and ≥50 years (Table 2). While the multivariable adjusted OR was 1.15 (p = 0.517), the matched patient population showed an estimated increased risk for diabetes in cohort B, OR = 1.47 (p = 0.089). We noted that the association of diabetes and age was not linear (Fig. 1) so the risk of diabetes by cohort was examined within age groups (age <50 and age ≥50). Both matched and unmatched analyses indicated that the risk of diabetes in cohort B vs. cohort A differed by age group. There was no significant difference in the risk between these two cohorts for age <50, but there was an increased risk for diabetes in cohort B vs. cohort A for age ≥50 (OR = 3.05 unmatched, 2.22 matched). The multivariable model of diabetes risk included an interaction term of cohort and age group, testing the premise that OR_age<50_ = OR_age≥50_ and estimated a significant difference between the odds ratios (p = 0.002), where OR_age<50_ represents the risk of diabetes in cohort B vs. A within age group<50. This result was consistent with prior studies that demonstrated an increased risk for recurrent TB in diabetic vs. non-diabetic TB patients although in our population this was restricted to the older age group [6].

**Table 2:** Rates of diabetes overall and by age group and cohort.

	Unadjusted diabetes rate (%)		p-value	Odds Ratio* [95% CI]
	Cohort A	Cohort B		Cohort B vs. Cohort A
**Overall**	52/220 (23.6)	110/437 (25.2)	0.517	1.15 [.75, 1.78]
▪ Age <50	34/140 (24.3)	53/302 (17.6)	0.269	0.75 [0.45,1.25]
▪ Age ≥50	18/80 (22.5)	57/135 (42.2)	0.003	3.05 [1.46, 6.36]
**Matched Cohorts** **	46/197 (23.4)	61/197 (31.0)	0.089	1.47 [0.95, 2.29]
▪ Matched, age <50	33/135 (24.4)	29/135 (21.5)	0.56	0.85 [0.48, 1.49]
▪ Matched, age ≥50	13/62 (20.9)	23/62 (37.1)	0.049	2.22 [1.00, 4.92]

* Adjusted for age, gender, smoking, drinking, education, occupation.

** Estimates and p-values based on generalized estimating equation regression.

### Characteristics on Presentation

At enrollment, radiographic measures and culture scores were compared (Table 3). Chest X-rays were read by radiologists blinded to patient status and graded according to a standardized scoring scheme. Comparing subjects with or without diabetes, there were no significant differences in rates of bilateral disease, cavitation or mid-lower lung zone opacities. There was, however, a significant difference in radiographic severity scores with non-diabetic subjects more likely than diabetic subjects to have radiographically minimal disease.

**Table 3:** Subject characteristics on enrollment.

		Non-Diabetic	Diabetic	p-value	
		n (%)	n (%)	Unadjusted	Adjusted*
**Radiographic severity**		495	162	0.04	0.03
	Minimal	18 (3.6)	1 (0.6)		
	Moderate	207 (41.8)	80 (49.4)		
	Advanced	270 (54.6)	81 (50.0)		
**Mid-lower opacities** **		388 (78.4)	138 (85.2)	0.07	0.23
**Bilateral lung disease**		414 (83.7)	137 (84.5)	0.90	0.78
**Cavitary lung disease**		304/419 (73.5)	100/134 (74.6)	0.82	0.91
**Smear Score**		495	162	0.52	0.38
	−	109 (22.0)	30 (18.5)		
	+	270 (54.6)	93 (57.4)		
	++	93 (18.8)	32 (19.8)		
	+++	23 (4.7)	7 (4.3)		
	++++	0	0		
**Culture Score**		495	158	0.92	0.90
	−	120 (24.2)	33 (20.9)		
	+	136 (27.5)	45 (28.5)		
	++	76 (15.4)	38 (24.1)		
	+++	160 (32.3)	42 (26.6)		
	++++	3 (0.6)	0		
**MDR-TB**		174 (40.8)	55 (40.2)	0.92	0.94

* Adjusted for cohort, age, gender, smoking.

** Opacities in the right middle lobe, right lower lobe, or left lower lobe.

There were no significant differences in sputum smear scores or culture scores, comparing diabetic with non-diabetic subjects. MDR-TB was identified at the time of enrollment in 16 of 192 (8.3%) of subjects in cohort A for whom data were available and 213/372 (57.3%) of subjects in cohort B. There was no significant difference in rates of MDR-TB comparing all diabetic to all non-diabetic subjects (Table 3), or when diabetic and non-diabetic subjects within cohort A or cohort B were compared individually (not shown). The high rate of MDR-TB in cohort B reflects that fact that these were retreatment cases.

### Response to Antimicrobial Treatment

Subjects in cohort A began treatment with isoniazid, rifampin, pyrazinamide and ethambutol (HRZE) with the intention to complete a 6–9 month standard short-course regimen. Any form of treatment for TB disease for >30 days prior to enrollment was an exclusion criterion for subjects in cohort A, making this cohort suitable for evaluation of sputum smear and culture conversion rates. Survival analysis was used to compare the rate of culture conversion after 2, 4 and 6 months of treatment. No statistically significant difference was observed between diabetic and non-diabetic subjects in cohort A (Table 4). Time to culture conversion was not evaluated in cohort B since many subjects had received antimicrobial treatment for variable intervals longer than 30 days before admission to NMTH and many were already on treatment and the time of enrollment in this study.

**Table 4:** Sputum culture conversion in cohort A.*

	2 Months	4 Months	6 Months
**% Negative Culture**			
▪ Diabetic (n = 44)	70.8%	90.3%	92.7%
▪ Non-Diabetic (n = 137)	70.8%	92.0%	97.3%

* Rates based on survival analysis; unadjusted p-value = 0.92; adjusted p-value = 0.98.

### Mortality Rates

**Figure 2:**
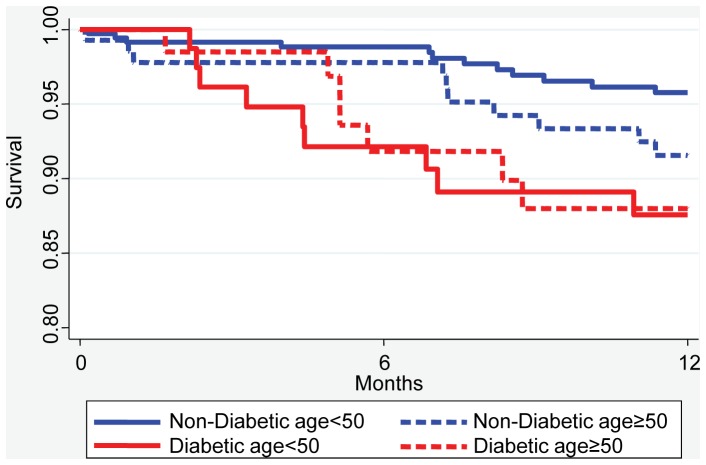
Survival estimates. Unadjusted Kaplan-Meier survival curve for all causes of death in diabetic subjects (*red lines*) and non-diabetic subjects (*blue lines*) grouped by age ≥50 (*dashed lines*) or age <50 (*solid lines*). Separate curves by age (dashed vs. solid) and diabetes (red vs. blue) illustrate the interaction of diabetes and age on survival. For age <50 the separation between diabetic vs. non-diabetic subjects is larger than for age ≥50.

Unadjusted survival was 87% in diabetics vs. 94% in non-diabetics during the first year after enrollment and TB treatment at NMTH. The adjusted hazard ratio (HR) for all-cause mortality in diabetic subjects compared with non-diabetic subjects, adjusted for cohort, age, gender and smoking was 2.18 (95% CI: 1.10, 4.34; Table 5). For TB-related deaths, the HR = 1.98 (95% CI: 0.90, 4.35; Table 6). Adjusting for MDR-TB in the all-cause or TB-related mortality models did not change the results appreciably, with HR = 2.40 (95% CI: 1.19, 4.2) for all-cause mortality and HR = 2.06 (95% CI: 0.94, 4.54) for TB-related deaths. This was unsurprising since drug resistance was highly correlated with cohort B that is included in the adjustment. MDR-TB status was missing in approximately 14% of the patients, so data presented in Tables 5 and 6 do not include the adjustment for MDR-TB. TB-related deaths in this study were attributable to respiratory failure, hemoptysis and to extrapulmonary TB (the latter reflecting only 2 of 49 TB-related deaths). Hemoptysis was the cause of death in the first year for 5 of 11 (46%) subjects with diabetes vs. 4 of 18 (22%) non-diabetic subjects, but this did not reach statistical significance. Figure 2 illustrates the unadjusted Kaplan-Meier estimates for all deaths, stratified by the presence or absence of diabetes and by age group. The survival difference between diabetic and non-diabetic subjects occurred mainly within the first 6 months of enrollment and treatment, after which the Kaplan-Meier curves were relatively parallel. The impact of diabetes on all-cause mortality was estimated to be greater in subjects <50 years of age (HR = 3.91 [95% CI: 1.54, 9.98]) than subjects ≥50 years old (HR = 1.12 [95% CI: 0.39, 3.18]). The test of interaction of diabetes and age resulted in p = 0.09 for all deaths. A similar interaction was estimated for TB-related deaths, with the impact of diabetes greater in subjects <50 years HR = 3.7 compared to the impact subjects >50 years, HR = 0.92 (p = 0.10; Fig. S1).

**Table 5:** Impact of diabetes and smoking on all cause one-year mortality.*

		All Deaths Unadjusted**		All Deaths Adjusted**	
		Hazard Ratio [95% CI]	p-value	Hazard Ratio [95% CI]	p-value
**Diabetic**	Non-diabetic	1***			
	Diabetic	2.41 [1.27, 4.59]	0.007	2.18 [1.10, 4.34]	0.026
**Smoker**	Nonsmoker	1		1	
	<1 pack	0.83 [0.34, 1.99]	0.67	1.46 [0.46, 4.66]	0.520
	≥1 pack	0.59 [0.27, 1.31]	0.20	1.95 [0.59, 6.46]	0.273
Cohort	A	1		1	
	B	2.96 [1.24, 7.07]	0.015	3.58 [1.36, 9.41]	0.010
Age	Years	1.03 [0.99, 1.07]	0.122	1.04 [1.00, 1.09]	0.067
Age ≥50	<50	1		1	
	≥50	0.83 [0.27, 2.51]	0.74	0.60 [0.19, 1.88]	0.384
Drinker	No	1		1	
	Yes	0.32 [0.16, 0.64]	0.001	0.36 [0.16, 0.78]	0.009
Sex	Male	1		1	
	Female	1.57 [0.72, 3.43]	0.257	2.19 [0.72, 6.67]	0.166
Education	Elem.	1		1	
	Middle	0.75 [0.29, 1.94]	0.553	0.67 [0.23, 1.96]	0.467
	High Sch	0.68 [0.28, 1.62]	0.381	0.79 [0.29, 2.14]	0.637
	Univ/Prof	1.03 [0.36, 2.96]	0.963	1.30 [0.39, 4.31]	0.670
Combined effects					
**Diabetic smoker**	<1 pack			3.19 [0.82, 12.38]	0.09
	≥1 pack			4.25 [1.06, 17.08]	0.04

* Smoking included a ‘missing’ group (not shown) in order to not lose data (5% missing); Age as continuous (linear) and an age indicator (<50 vs. ≥50) were included together for unadjusted age association. Combined Diabetes and smoker are additive main effects (not an interaction); comparison of diabetic smoker vs. non-diabetic non-smoker.

** The column All Deaths Unadjusted shows estimated unadjusted associations of each factor with death. The column All Deaths Adjusted shows estimated adjusted associations from a single model using all the factors in the table (each factor is adjusted for all other factors in that table). The combined effect at the bottom is a combination of coefficients for diabetes and smoking from the adjusted model.

*** Reference value.

**Table 6:** Impact of diabetes and smoking on one-year mortality attributable to TB.*

		TB Deaths Unadjusted**		TB Deaths Adjusted**	
		Hazard Ratio [95% CI]	p-value	Hazard Ratio [95% CI]	p-value
**Diabetic**	Non-diabetic	1***		1	
	Diabetic	2.02 [0.95, 4.27]	0.067	1.98 [0.90, 4.35]	0.091
**Smoker**	Nonsmoker	1		1	
	<1 pack	1.10 [0.38, 3.18]	0.855	2.10 [0.53, 8.30]	0.29
	≥1 pack	0.79 [0.30, 2.11]	0.395	2.93 [0.70, 12.28]	0.14
Cohort	A	1		1	
	B	3.45 [1.20, 9.92]	0.022	3.48 [1.18, 10.27]	0.024
Age	Years	1.02 [0.98, 1.07]	0.309	1.04 [0.99, 1.09]	0.125
Age ≥50	<50	1		1	
	≥50	0.86 [0.24, 3.06]	0.813	0.60 [0.17, 2.16]	0.431
Drinker	No	1		1	
	Yes	0.31 [0.14, 0.69]	0.004	0.32 [0.13, 0.77]	0.431
Sex	Male	1		1	
	Female	1.53 [0.62, 3.75]	0.355	3.03 [0.87, 10.51]	0.081
Education	Elem.	1		1	
	Middle	0.89 [0.31, 2.57]	0.830	0.75 [0.23, 2.45]	0.627
	High Sch	0.71 [0.26, 1.92]	0.501	0.74 [0.24, 2.30]	0.599
	Univ/Prof	0.91 [0.26, 3.22]	0.880	1.05 [0.25, 4.36]	0.943
Combined effects					
**Diabetic smoker**	<1 pack			4.14 [0.83, 20.71]	0.08
	≥1 pack			5.78 [1.09, 30.56]	0.04

* Smoking included a ‘missing’ group (not shown) in order to not lose data (5% missing); Age as continuous (linear) and an age indicator (<50 vs. ≥50) were included together for unadjusted age association. Combined Diabetes and smoker are additive main effects (not an interaction); comparison of diabetic smoker vs. non-diabetic non-smoker.

** The column TB Deaths Unadjusted shows estimated unadjusted associations of each factor with death. The column TB Deaths Adjusted shows estimated adjusted associations from a single model using all the factors in the table (each factor is adjusted for all other factors in that table). The combined effect at the bottom is a combination of coefficients for diabetes and smoking from the adjusted model.

*** Reference value.

In the adjusted models there was an estimated increase in risk of mortality for smokers vs. nonsmokers for all causes of death and for TB-related death. Of note, only 20% of diabetic subjects were self-described non-smokers while a majority (54%) admitted to smoking ≥1 pack of cigarettes daily prior to enrollment. No patients were permitted to smoke after admission to NMTH. Interaction of diabetes and smoking was not significant (p>0.50 in all cases). The hazard for diabetics who also smoke cigarettes could therefore be estimated by combining the two risks (estimated risk in a diabetic, ≥ one pack smoker vs. non-diabetic, non-smoker). For all deaths, HR = 4.25 [95% CI: 1.06, 17.08] and for TB-related deaths: 5.78 [95% CI: 1.09, 30.56].

## Discussion

Despite its advanced economy, the Republic of Korea has relatively high TB incidence (97/100,000 in 2006) [13]. The prevalence of diabetes is also high in Korea, estimated at 9.1% in 2005 as compared to <1% in 1970 [14]. It is therefore important to understand the impact that diabetes has on the manifestations and outcomes of TB disease in the Korean population, and its relationship to similar data from other populations. Our investigation made use of a large, prospectively recruited cohort of TB patients at NMTH. The 25% prevalence of diabetes in this TB cohort is in line with the prevalence of diabetes in the general Korean population and an approximately 3-fold increased TB risk for people with diabetes reported in previous studies [5]. Results in the current study were consistent with an adverse impact of diabetes on TB disease severity, reflected by increased mortality at one year.

A recent meta-analysis of evidence pertaining to the impact of diabetes on TB outcomes identified four studies where mortality data were adjusted for age and certain other potential confounders [6]. These include a retrospective review by Fielder et al. [15] of 174 subjects with TB reported to the Baltimore City Health Department between 1993 and 1998, that calculated an adjusted OR for death in the 22 diabetic subjects of 3.8 (95% CI:1.4, 10.3). The median time to death after starting TB treatment was 32 days and the majority of deaths were due to causes other than TB. Oursler et al. [16] also retrospectively evaluated 139 subjects with pulmonary TB reported to the Baltimore City Health Department between 1994 and 1996. Eighteen subjects (14%) had diabetes with adjusted HR for mortality of 6.7 (95% CI:1.6, 29.3). Reflecting that this was a subset of the same population reported by Fielder, the overall mortality was 21% with a median time to death of 39 days. The third published study reporting adjusted mortality data in TB patients was conducted retrospectively in cases of culture-confirmed disease in Montgomery County, Prince George’s county and Baltimore City in Maryland, USA [17]. Overall mortality for the 217 subjects was 9%. Mortality in the 42 subjects (14%) with diabetes was higher than non-diabetic subjects (OR = 6.5; 95% CI: 1.11, 38.20). Drug-resistant bacilli were found on initial culture in a similar proportion of diabetic and non-diabetic subjects (14.1% and 15.1%, respectively), none of the 26 deaths occurred in subjects with MDR-TB and there was no acquired drug resistance was recorded. Finally, Wang et al. [18] retrospectively reviewed 271 culture-positive pulmonary TB patients at the Kaohsiung Municipal Hsiao-Kang Hospital in Taiwan between 2003 and 2006, of whom 74 (34%) were diabetic. All-cause mortality at one year in the diabetic group was 12.2% compared to 4.2% in non-diabetic subjects (OR = 7.6 adjusted for age and gender; 95% CI: 1.976, 29.083). The mean age of diabetic and non-diabetic subjects in that study (60.8 and 59.1) was nearly two decades older than our cohort, and they found that older age was an independent predictive factor for unfavorable outcomes.

Our findings in 557 prospectively recruited Koreans with TB disease were generally consistent with the outcome studies cited above, albeit with some qualitative and quantitative differences that may reflect the geographic location and demographics of the populations studied. The reports of Fielder, Oursler, and Dooley used data collected in Baltimore or adjacent counties in Maryland and those of Fielder and Oursler appear to have analyzed a largely overlapping set of records. All three studies reported strikingly high early mortality. The impact of diabetes on mortality risk in our cohort was quantitatively more comparable to summary RR 1.89 for all 23 studies reviewed by Baker et al. [6], most being unadjusted. These differences in overall mortality for our study vs. the four studies with adjusted data may reflect the high proportion of subjects co-infected with HIV (24%) in the cohorts of Fielder and Oursler, and the older age of the population reported by Wang. In a separate report of 157 TB cases collected at the Kaohsiung Municipal Hsiao-Kang Hospital in Taiwan between 2003 and 2006, Wang et al. [19] focused on the impact of age on outcomes. All-cause mortality in subjects ≥60 years old (median age 74) was 26.5% in contrast to 4.1% for younger patients (median age 46) despite comparable frequencies of diabetes in the two groups (32.5% and 29.7%, respectively). Deaths in diabetic vs. non-diabetic subjects within the two age groups were not described in that report. In contrast to the studies from Taiwan and Baltimore, relative youth had no evident protective effect for all-cause mortality in our cohort where 21.3% of diabetic subjects ≥50 and 17.2% of diabetic subjects <50 died within one year of enrollment.

Our study also differed from those of Fielder, Oursler, Dooley and Wang by considering the impact of smoking on TB mortality. This question was recently investigated in a 14-year prospective cohort of 1,294,504 Koreans aged 30–95 who participated in a biennial National Health Insurance Corporation medical evaluation [4]. Smoking was associated with increased mortality from TB in men and women, with similar risks for current and former smokers and no indication of increasing risk based on the number of cigarettes smoked daily. For male current smokers vs. non-smokers in that study, HR = 1.58 (95% CI: 1.27, 1.97). Smoking and diabetes are highly prevalent conditions that increase the risk of developing active TB and the risk of adverse outcomes from TB disease [4], [5]. We confirmed the lack of interaction between smoking and diabetes in our cohort and found that the combination of smoking and diabetes significantly increased the hazard of death from all causes and death attributable to TB disease in comparison to non-diabetic, non-smokers. This adverse impact of smoking history was observed despite the fact that subjects were not permitted to smoke while inpatients at NMTH.

Our investigation of TB outcomes had several limitations. The study population was biased for greater TB severity with a large number of retreatment cases (cohort B). This could reduce any detectable impact of diabetes on outcomes. Arguing against that, we found a similar impact of diabetes on mortality in cohorts A and B. Our analysis of mortality was hampered by the fact that the cause of death information was unavailable for one quarter of the subjects who expired. The variable initiation of TB treatment prior to enrollment in cohort B made it impossible to reliably track time to sputum conversion in that group. Finally, the original design of the Natural History Study from which data were extracted was not focused on diabetes. While subjects could be identified as having diabetes or not, the duration and severity diabetes in individual subjects was unknown. This is a major limitation for many studies of this topic. Limited clinical information and results from the mouse model suggest that like most other complications of diabetes, TB susceptibility results from a cumulative impact of chronic hyperglycemia [20]–[23]. While the possible correlation of diabetes severity with TB outcomes has not been directly tested, Baker et al. [24] recently reported that TB disease risk is increases as the number of diabetes-related complication increases.

Our study adds to a growing body of evidence that diabetes mellitus not only increases the risk of developing active TB but also increases the severity of TB disease, reflected by higher risk of recurrent TB and death. We further show that diabetes and smoking are independent risks for adverse outcomes with TB disease that in combination significantly increase the hazard of TB-related death. This is concerning in view of the rising rates of diabetes and tobacco use in the emerging economies of Asia where TB is already prevalent. Also concerning in our results was the finding that diabetes was associated with increased mortality in subjects <50 years old. Evidence that diabetes and smoking contribute significantly to TB disease risk and severity raises the question whether current public health and TB control practices adequately address the problem. Presently, diabetic patients with drug sensitive TB are treated with short course HRZE. The increased risk of relapse in this population suggests the need to test the efficacy of prolonging the consolidation phase of treatment in patients with diabetes. Similarly, the increased risk of death during TB treatment might be addressed by intensifying management and observation in patients with diabetes, particularly those who smoke. The combined impact of diabetes and smoking on TB disease risk and mortality may provide a rationale to treat latent TB infection in those at risk, which is not current clinical practice in low and middle income countries [25]. An interventional trial of LTBI treatment in diabetics would be necessary to confirm safety and efficacy in that population.

## Supporting Information

Figure S1 Adjusted Diabetes*age interaction model. Estimated survival curves based on adjusted Cox regression models for all-cause mortality (A) and TB-related mortality (B) in diabetic subjects (*red lines*) and non-diabetic subjects (*blue lines*) stratified by age ≥50 (*dashed lines*) or age <50 (*solid lines*). The impact of diabetes (red vs. blue lines) is greater in subjects age <50 years old.
(PDF)

## References

[1] Lonnroth K, Castro KG, Chakaya JM, Chauhan LS, Floyd K, et al (2010) Tuberculosis control and elimination 2010–50: cure, care, and social development. Lancet 375: 1814–1829. 20488524 10.1016/S0140-6736(10)60483-7

[2] Getahun H, Gunneberg C, Granich R, Nunn P (2010) HIV infection-associated tuberculosis: the epidemiology and the response. Clin Infect Dis 50 Suppl 3S201–S207. 20397949 10.1086/651492

[3] Restrepo BI, Fisher-Hoch SP, Crespo JG, Whitney E, Perez A, et al (2007) Type 2 diabetes and tuberculosis in a dynamic bi-national border population. Epidemiol Infect 135: 483–491. 16863600 10.1017/S0950268806006935 PMC2870584

[4] Jee SH, Golub JE, Jo J, Park IS, Ohrr H, et al (2009) Smoking and risk of tuberculosis incidence, mortality, and recurrence in South Korean men and women. Am J Epidemiol 170: 1478–1485. 19917554 10.1093/aje/kwp308 PMC2800271

[5] Jeon CY, Murray MB (2008) Diabetes mellitus increases the risk of active tuberculosis: a systemic review of 13 observational studies. PLoS Med 5: e152. 18630984 10.1371/journal.pmed.0050152 PMC2459204

[6] Baker MA, Harries AD, Jeon CY, Hart JE, Kapur A, et al (2011) The impact of diabetes on tuberculosis treatment outcomes: a systematic review. BMC Med 9: 81. 21722362 10.1186/1741-7015-9-81 PMC3155828

[7] Yim JJ, Selvaraj P (2010) Genetic susceptibility in tuberculosis. Respirology 15: 241–256. 20199642 10.1111/j.1440-1843.2009.01690.x

[8] Lee JW, Brancati FL, Yeh HC (2011) Trends in the prevalence of type 2 diabetes in Asians versus whites: results from the United States National Health Interview Survey, 1997–2008. Diabetes Care 34: 353–357. 21216863 10.2337/dc10-0746 PMC3024348

[9] Shamputa IC, Lee J, Allix-Beguec C, Cho EJ, Lee JI, et al (2010) Genetic diversity of Mycobacterium tuberculosis isolates from a tertiary care tuberculosis hospital in South Korea. J Clin Microbiol 48: 387–394. 20018816 10.1128/JCM.02167-09 PMC2815581

[10] Long ED, Hopkins FD (1952) History of diagnostic standards and classification of tuberculosis of the National Tuberculosis Association. Am Rev Tuberc 65: 494–504. 14903517 10.1164/art.1952.65.4.494

[11] Cleveland WS (1993) Bivariate Data. In: Visualizing Data. Summit, NJ: Hobart Press. 86–179.

[12] Wang JY, Lee LN, Lai HC, Hsu HL, Liaw YS, Hsueh PR, Yang PC (2007) Prediction of the tuberculosis reinfection proportion from the local incidence. J Infect Dis 196: 281–288. 17570116 10.1086/518898

[13] World Health Organization (2011) Global Tuberculosis Control: WHO report 2011. Geneva, Switzerland: World Health Organization. Publication no. WHO/HTM/TB/2011.16.

[14] Choi YJ, Kim HC, Kim HM, Park SW, Kim J, et al (2009) Prevalence and management of diabetes in Korean adults: Korea National Health and Nutrition Examination Surveys 1998–2005. Diabetes Care 32: 2016–2020. 19675201 10.2337/dc08-2228 PMC2768204

[15] Fielder JF, Chaulk CP, Dalvi M, Gachuhi R, Comstock GW, et al (2002) A high tuberculosis case-fatality rate in a setting of effective tuberculosis control: implications for acceptable treatment success rates. Int J Tuberc Lung Dis 6: 1114–1117. 12546121

[16] Oursler KK, Moore RD, Bishai WR, Harrington SM, Pope DS, et al (2002) Survival of patients with pulmonary tuberculosis: clinical and molecular epidemiologic factors. Clin Infect Dis 34: 752–759. 11850859 10.1086/338784

[17] Dooley KE, Tang T, Golub JE, Dorman SE, Cronin W (2009) Impact of diabetes mellitus on treatment outcomes of patients with active tuberculosis. Am J Trop Med Hyg 80: 634–639. 19346391 PMC2750857

[18] Wang CS, Yang CJ, Chen HC, Chuang SH, Chong IW, et al (2009) Impact of type 2 diabetes on manifestations and treatment outcome of pulmonary tuberculosis. Epidemiol Infect 137: 203–210. 18559125 10.1017/S0950268808000782

[19] Wang CS, Chen HC, Yang CJ, Wang WY, Chong IW, et al (2008) The impact of age on the demographic, clinical, radiographic characteristics and treatment outcomes of pulmonary tuberculosis patients in Taiwan. Infection 36: 335–340. 18629436 10.1007/s15010-008-7199-8

[20] Martens GW, Arikan MC, Lee J, Ren F, Greiner D, et al (2007) Tuberculosis susceptibility of diabetic mice. Am J Respir Cell Mol Biol 37: 518–524. 17585110 10.1165/rcmb.2006-0478OC PMC2048677

[21] Restrepo BI, Fisher-Hoch SP, Pino PA, Salinas A, Rahbar MH, et al (2008) Tuberculosis in poorly controlled type 2 diabetes: altered cytokine expression in peripheral white blood cells. Clin Infect Dis 47: 634–641. 18652554 10.1086/590565 PMC2900313

[22] Leung CC, Lam TH, Chan WM, Yew WW, Ho KS, et al (2008) Diabetic control and risk of tuberculosis: a cohort study. Am J Epidemiol 167: 1486–1494. 18400769 10.1093/aje/kwn075

[23] Park SW, Shin JW, Kim JY, Park IW, Choi BW, et al (2012) The effect of diabetic control status on the clinical features of pulmonary tuberculosis. Eur J Clin Microbiol Infect Dis 31: 1305–1310. 22042559 10.1007/s10096-011-1443-3

[24] Baker MA, Lin H-H, Chang H-Y, Murray MB (2012) The effect of diabetic control status on the clinical features of pulmonary tuberculosis. Clin Infect Dis 53: 818–825.

[25] Menzies D, Al JH, Al OB (2011) Recent developments in treatment of latent tuberculosis infection. Indian J Med Res 133: 257–266. 21441678 PMC3103149

