# Supplementary figures and images for: Impact of Diabetes and Smoking on Mortality in Tuberculosis
Source: PLoS One. 2013 Feb 28;8(2):e58044. doi: 10.1371/journal.pone.0058044 (PMC3585219; doi:10.1371/journal.pone.0058044)

Figure S1. Adjusted Diabetes\*age interaction model.

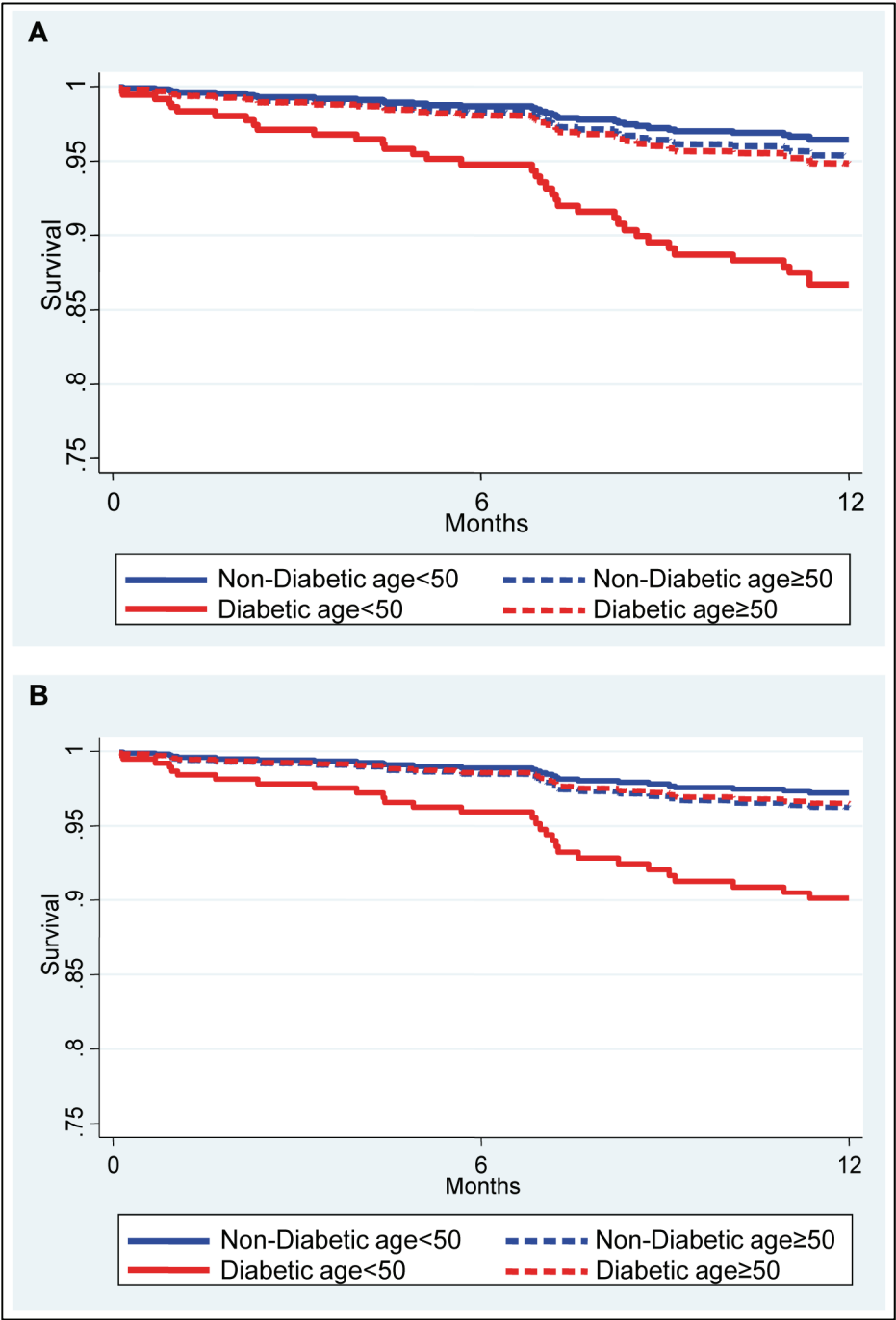

Supplement: Figure S1 — Adjusted Diabetes*age interaction model. Estimated survival curves based on adjusted Cox regression models for all-cause mortality (A) and TB-related mortality (B) in diabetic subjects (red lines) and non-diabetic subjects (blue lines) stratified by age ≥50 (dashed lines) or age <50 (solid lines). The impact of diabetes (red vs. blue lines) is greater in subjects age <50 years old. (PDF) [file pone.0058044.s001.pdf]
